# Bafilomycin 1A Affects p62/SQSTM1 Autophagy Marker Protein Level and Autophagosome Puncta Formation Oppositely under Various Inflammatory Conditions in Cultured Rat Microglial Cells

**DOI:** 10.3390/ijms25158265

**Published:** 2024-07-29

**Authors:** István Pesti, Gábor Barczánfalvi, Karolina Dulka, Diana Kata, Eszter Farkas, Karoly Gulya

**Affiliations:** 1 Department of Cell Biology and Molecular Medicine, University of Szeged, 6720 Szeged, Hungary; pesti.istvan@med.u-szeged.hu (I.P.); barczanfalvi.gabor@med.u-szeged.hu (G.B.); karolina.dulka@med.u-szeged.hu (K.D.); farkas.eszter1@med.u-szeged.hu (E.F.); 2 HCEMM-USZ Group of Cerebral Blood Flow and Metabolism, University of Szeged, 6720 Szeged, Hungary; 3 Department of Laboratory Medicine, University of Szeged, 6725 Szeged, Hungary; kata.diana@med.u-szeged.hu

**Keywords:** autophagosome, autophagy, bafilomycin A1, inflammation, lipopolysaccharide, microglia, p62/SQSTM1, rosuvastatin

## Abstract

Regulation of autophagy through the 62 kDa ubiquitin-binding protein/autophagosome cargo protein sequestosome 1 (p62/SQSTM1), whose level is generally inversely proportional to autophagy, is crucial in microglial functions. Since autophagy is involved in inflammatory mechanisms, we investigated the actions of pro-inflammatory lipopolysaccharide (LPS) and anti-inflammatory rosuvastatin (RST) in secondary microglial cultures with or without bafilomycin A1 (BAF) pretreatment, an antibiotic that potently inhibits autophagosome fusion with lysosomes. The levels of the microglia marker protein Iba1 and the autophagosome marker protein p62/SQSTM1 were quantified by Western blots, while the number of p62/SQSTM1 immunoreactive puncta was quantitatively analyzed using fluorescent immunocytochemistry. BAF pretreatment hampered microglial survival and decreased Iba1 protein level under all culturing conditions. Cytoplasmic p62/SQSTM1 level was increased in cultures treated with LPS+RST but reversed markedly when BAF+LPS+RST were applied together. Furthermore, the number of p62/SQSTM1 immunoreactive autophagosome puncta was significantly reduced when RST was used but increased significantly in BAF+RST-treated cultures, indicating a modulation of autophagic flux through reduction in p62/SQSTM1 degradation. These findings collectively indicate that the cytoplasmic level of p62/SQSTM1 protein and autophagocytotic flux are differentially regulated, regardless of pro- or anti-inflammatory state, and provide context for understanding the role of autophagy in microglial function in various inflammatory settings.

## 1. Introduction

Microglia are macrophage-like resident immune cells in the brain that are activated in response to various factors, including cytokines, chemokines, nitric oxide, reactive oxygen intermediates, and various stimuli of neuropathological origin, including trauma, stroke, and infection [1]. Under physiological conditions, microglial cells contribute to the maintenance and resolution of brain homeostasis. In pathological states, they release high levels of pro-inflammatory mediators and cytotoxic factors that activate nearby microglia, which can propagate neuroinflammation and degeneration [2]. Microglia also exert neuroprotective effects, depending on their various functional phenotypes in response to specific stimuli through the production of anti-inflammatory factors [3]. For example, up-regulation of autophagy stimulates microglia to produce anti-inflammatory factors, while inhibition of autophagy results in the release of inflammatory cytokines [4].

The ubiquitin-binding protein (p62), also known as autophagosome cargo protein sequestosome 1 (SQSTM1), is a stress-inducible scaffold protein crucial for various cellular functions such as autophagy, apoptosis, inflammation, cell survival/death, signal transduction, and tumorigenesis [5,6]. The protein p62/SQSTM1 localizes at the autophagosome formation site on the endoplasmic reticular membrane and recognizes toxic cellular waste that is then scavenged by a sequestration process known as autophagy [7]. Because p62/SQSTM1 serves as a substrate for autophagic degradation, its level is inversely proportional to autophagic activity [8,9,10]. Degradation of p62/SQSTM1 may link ubiquitinated proteins to the autophagic machinery to enable their degradation in the lysosome [11]. On the contrary, reduced autophagy leads to the accumulation of p62/SQSTM1 that induces a cellular stress response that leads to disease [9,12,13], promotes aging [14], and could also be involved in neurodegenerative processes [15,16].

Statins (3-hydroxy-3-methylglutaryl coenzyme A reductase inhibitors) are the agents of choice for the treatment of high blood cholesterol levels [17]. Although their main effects are related to lipid metabolism, they also strongly modulate inflammatory cells around atherosclerotic plaques. Rosuvastatin ((E)-7-[4-(4-Fluorophenyl)-6-isopropyl-2-[methyl(methylsulfonyl)amino]pyrimidin-5-yl]-(3R,5S)-3,5-dihydroxyhept-6-enoic acid); RST) is one of the most prescribed drugs, with the most favorable metabolic and atherosclerotic plaque-clearing profile among statins [17,18]. In addition to its therapeutic use in cardiovascular diseases, RST also has beneficial effects in attenuating inflammatory responses both in vitro [3] and in vivo [19].

Previous studies suggested that autophagy is associated with controlling macrophage activation and survival [20]. For example, inflammatory signals reciprocally control autophagy [21,22], which, in turn, plays an anti-inflammatory role and suppresses pro-inflammatory processes by regulating innate immune signaling pathways [23]. One of the drugs that interferes with autophagic flux is the macrolide antibiotic bafilomycin A1 ((3Z,5E,7R,8S,9S,11E,13E,15S,16R)-16-[(1S,2R,3S)-3-[(2R,4R,5S,6R)-2,4-dihydroxy-6-isopropyl-5-methyl-2-tetrahydropyranyl]-2-hydroxy-1-methylbutyl]-8-hydroxy-3,15-dimethoxy-5,7,9,11-tetramethyl-1-oxacyclohexadeca-3,5,11,13-tetraen-2-one; BAF). It is a potent inhibitor of the vacuolar type H^+^-ATPase [24,25]. BAF inhibits autophagic flux by preventing endosome and lysosome acidification [26,27] and autophagic vacuole maturation by inhibiting fusion between autophagosomes and lysosomes [28], resulting in the fact that autophagic substrates cannot be degraded in the lysosome.

Although previous studies showed that p62/SQSTM1 levels increased in activated microglia [2], the mechanisms by which inflammatory signals specifically relieve autophagy suppression of inflammation, or the precise role that p62/SQSTM1-mediated autophagic regulatory mechanism plays in microglial function, are not clearly understood. Our hypothesis was that BAF will interfere with autophagocytotic processes in microglia regardless of different inflammatory conditions. In this study, we set out to localize and quantitatively analyze cytoplasmic p62/SQSTM1 levels together with p62/SQSTM1 immunoreactive puncta of p62/SQSTM1 in various pro- and anti-inflammatory settings using bacterial lipopolysaccharide (LPS) and RST, respectively, in the presence or absence of BAF in secondary microglial cultures. Our findings further contribute to understanding the mechanistic intricacies of autophagy regulation in the context of pro- and anti-inflammatory stimuli in microglial cultures.

## 2. Results

### 2.1. BAF Inhibits Microglia Proliferation

**Figure 1:**
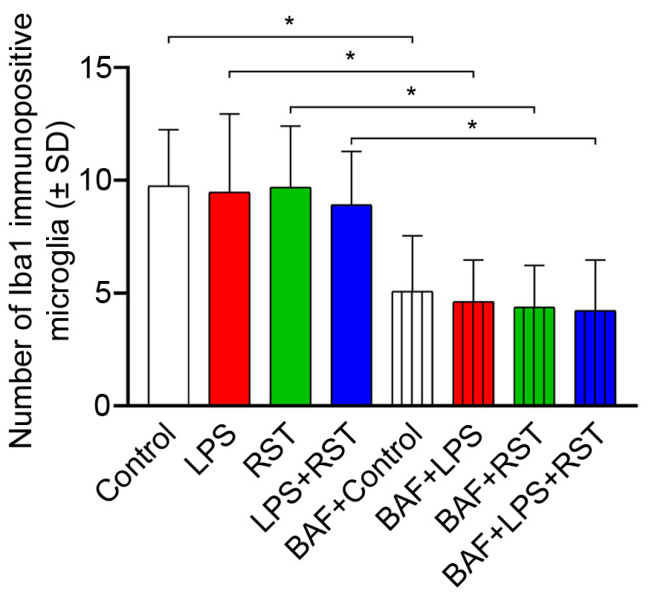
Effect of treatments on the number of Iba1 immunopositive microglial cells in cultures (subDIV7). The number of Iba1-positive microglial cells in control, LPS-, RST- and LPS+RST-treated cultures with or without BAF pretreatment was counted and averaged (±SD) according to the protocols described in Materials and Methods. Treatments of the cultures (in final concentrations) were as follows: LPS = 20 ng/mL; RST = 1 μM; LPS+RST = 20 ng/mL + 1 μM; BAF = 50 nM). The normality of the data distribution was determined using a Shapiro–Wilk test. Data were analyzed using one-way analysis of variance (ANOVA), followed by Tukey’s multiple comparison. * denotes significant differences (*p* < 0.05) between samples with or without BAF pretreatment.

There were no statistically significant differences in the number of microglial cells among different treatment regimens without pretreatment with the autophagocytosis inhibitor BAF (Figure 1). However, as expected, pretreatment with BAF significantly reduced microglia proliferation by approximately 45–50% (*p* < 0.05) in all treatment groups investigated compared to cultures without BAF pretreatment. Furthermore, no significant differences were found in the number of microglial cells within the BAF-pretreated groups.

### 2.2. Pro- and Anti-Inflammatory Drugs Affect Soluble Iba1 and p62/SQSTM1 Protein Contents Differently

**Figure 2:**
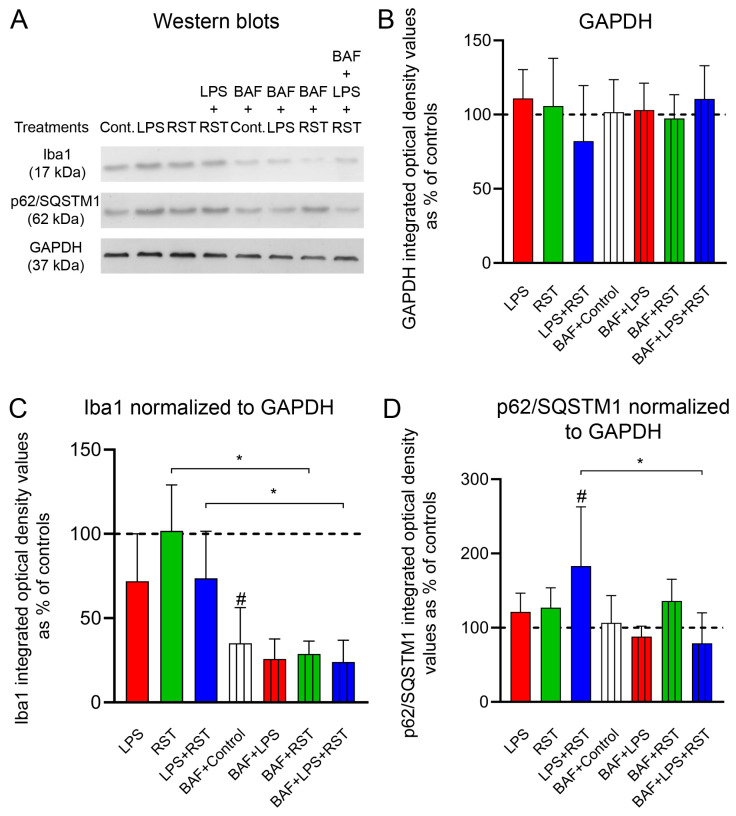
Quantitative Western blot analyses of Iba1 and p62/SQSTM1 immunoreactivities from secondary microglia cultures (subDIV7). (**A**) Representative western blot images of p62/SQSTM1, Iba1 and GAPDH immunoreactivities in secondary microglial cell cultures. (**B**) Quantitative Western blot analysis of GAPDH immunoreactivities in control and treated microglial cell cultures. (**C**) Quantitative western blot analysis of Iba1 immunoreactivities in secondary microglial cell cultures normalized for GAPDH immunoreactivity. (**D**) Quantitative western blot analysis of p62/SQSTM1 immunoreactivities in secondary microglial cell cultures normalized for GAPDH immunoreactivity. The error bars indicate the integrated optical density values as % of the controls (mean ± SD of at least 4 separate experiments). The normality of the data distribution was determined using a Shapiro–Wilk test. Data were analyzed using ANOVA, followed by Tukey’s multiple comparison. # denotes significant differences between control and treated cultures. * denotes significant differences (*p* < 0.05) between samples with or without BAF pretreatment.

The total cellular soluble protein content under different culture conditions in the absence or presence of BAF was quantitatively analyzed on Western blots (Figure 2, Supplementary Table S1). Iba1 protein content was somewhat sensitive to both pro-inflammatory LPS treatment alone and combined treatment with LPS+RST, although these inhibitory effects did not reach the significance level (Figure 2A,C). However, the total amount of soluble Iba1 was sensitive to BAF pretreatment, when 50–60% decreases were detected in each experimental group compared to controls without BAF (Figure 2A,C). Specifically, Iba1 protein levels were significantly reduced in controls pretreated with BAF (# *p* < 0.05), BAF-pretreated RST-treated (* *p* < 0.05), and BAF-pretreated LPS+RST-treated cultures (* *p* < 0.05) as compared to those respective cultures without BAF pretreatment.

The soluble component of the p62/SQSTM1 pool was less sensitive to treatments as compared to Iba1 levels (Figure 2D vs. Figure 2C). Although all treatments without BAF pretreatment increased cytoplasmic p62/SQSTM1 immunoreactivities, only the combined treatment of LPS+RST resulted in a significant increase (# *p* < 0.05) over the control level, indicating a strong inhibition of autophagy under such circumstances (Figure 2A,D). However, when this elevated p62/SQSTM1 level in the LPS+RST-treated culture was compared to the levels found in BAF+LPS+RST cultures (Figure 2D), a significant decrease in p62/SQSTM1 immunoreactivity was detected (* *p* < 0.05). Other treatments combined with BAF pretreatment did not have a significant effect on the amount of this protein. Finally, GAPDH, a protein considered a housekeeping enzyme with a stable rate of synthesis, did not change significantly in any of these groups (Figure 2A,B).

### 2.3. Pro- and Anti-Inflammatory Drugs Affect Iba1 and p62/SQSTM1 Immunocytochemical Signals Differently

**Figure 3:**
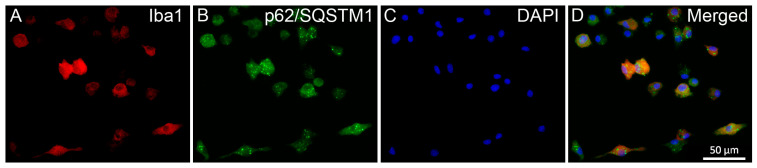
Localization of p62/SQSTM1 immunoreactivities in unchallenged and untreated secondary microglial cultures (subDIV7). (**A**) Microglia (red) were detected by incubating cultures with anti-Iba1 antibody). (**B**) Autophagosomes were detected by incubating cells with anti-p62/SQSTM1 antibody (green). p62/SQSTM1 immunopositivity was found in the highest number around the nucleus. (**C**) Nuclei were stained with DAPI (blue). (**D**) Merged images. Scale bar in (**D**) (for all pictures): 50 µm.

**Figure 4:**
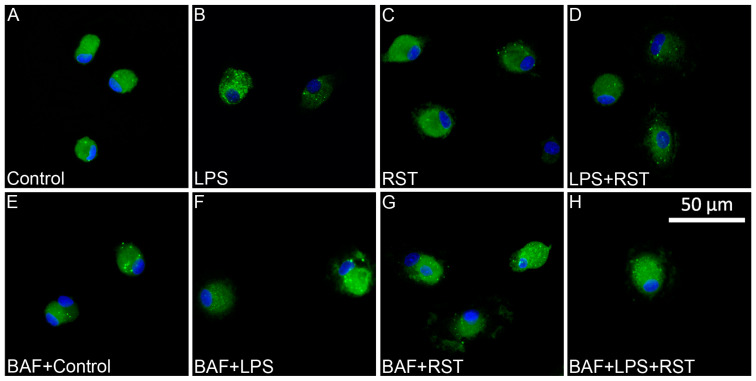
Representative fluorescent immunocytochemical images of microglia containing p62/SQSTM1-immunolabeled autophagosomes. (**A**) Control (unchallenged and untreated), (**B**) LPS-challenged, (**C**) RST-treated, (**D**) LSP-challenged + RST-treated, (**E**) BAF pretreated + control (unchallenged and untreated), (**F**) BAF pretreated + LPS-challenged, (**G**) BAF pretreated + RST-treated and (**H**) BAF pretreated + LSP-challenged + RST-treated microglial cells were analyzed. The p62/SQSTM1 protein was detected by incubating first with mouse anti-p62/SQSTM1 primary antibody followed by goat anti-mouse secondary antibody conjugated to Alexa Fluor 488 fluorochrome (green), while microglia nuclei are labeled with DAPI (blue). The cytoplasm of the microglia contains several p62/SQSTM1-labeled puncta. Scale bar in (**H**) (for all pictures): 50 µm.

The effects of different inflammatory states on the distribution of p62/SQSTM1 immunoreactivity were analyzed in representative multicolor fluorescent immunocytochemical pictures (Figure 3 and Figure 4) of control and treated microglia cultures (LPS, RST, or LPS+RST) in the absence (Supplementary Figure S1) or presence of BAF (Supplementary Figure S2). Microglial cells (subDIV7), identified by their Iba1 immunopositivity (Figure 3A), showed p62/SQSTM1 immunoreactivity both in the cytosol and compartmentalized in autophagosomes (Figure 3B,D). p62/SQSTM1 immunoreactivity was evenly distributed in the cytoplasm, while p62/SQSTM1-labeled autophagosomes were predominantly located as puncta in the perinuclear cytoplasm and rarely in microglial processes (Figure 3B).

Different treatments affected the amount of p62/SQSTM1 immunoreactivity in microglial cells differently. Fluorescent light microscopic localization of p62/SQSTM1-positive phagosomes revealed two notable characteristics in terms of their responses to treatment. We found a similar number of p62/SQSTM1-labeled autophagosomes in cultures challenged with LPS but found significantly less puncta in RST-treated cultures (Figure 4); however, when RST-treated cultures were pretreated with BAF, this effect was reversed and the number of p62/SQSTM1-positive phagosomes increased.

### 2.4. BAF Alters Autophagocytotic Puncta Formation and Affects Autophagic Flux Only in Cultures Treated with RST

**Figure 5:**
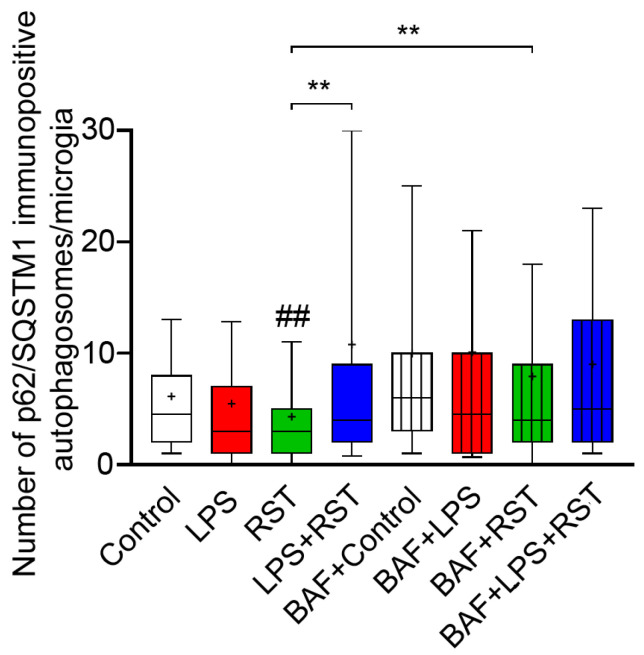
Quantitative analysis of p62/SQSTM1-labeled autophagosomes in microglial cultures (subDIV7) with or without BAF pretreatment. The number of p62/SQSTM1 immunopositive phagosomes per Iba1-positive microglia was analyzed in control (unchallenged), LPS-challenged, LPS-challenged and RST-treated cultures with or without BAF pretreatment. Data are presented as 10 percentile to 90 percentile. The normality of the data distribution was determined using a Shapiro–Wilk test. Data were analyzed using a Kruskal–Wallis test, followed by Dunn’s multiple comparison. ## *p* < 0.01 vs. control; ** *p* < 0.01. The mean and median with 10–90 percentiles are visualized in the box plot. + denotes the mean value. The median value is indicated as a horizontal line inside the box.

There was a significant decrease (## *p* < 0.01) in puncta formation when RST was applied to the cultures as compared to the control levels (Figure 5, Supplementary Table S2). However, we found significantly (** *p* < 0.01) elevated p62/SQSTM1-immunolabeled puncta formation in microglia only when the combined treatment of BAF+RST was applied to the cultures as compared to the levels observed with RST treatment alone. Interestingly, combined treatment with LPS+RST also significantly (** *p* < 0.01) increased autophagosome formation when compared to the decreased levels observed with RST treatment alone.

## 3. Discussion

Effective elimination of harmful material from the cytoplasm is essential to maintain cellular homeostasis and is achieved primarily by autophagy [12]. Under various conditions, autophagy prevents cell damage and promotes survival by the formation of a cytosolic double-membrane vesicle, the autophagosome [29]. In mammals, autophagy often converges with the endocytic pathway, as autophagosomes fuse with lysosomes to form autolysosomes, in which resident hydrolases degrade cytoplasmic cargoes [30,31].

Microglial activation, as a central component of innate immunity in the nervous system, is a characteristic feature of neurodegenerative diseases and neuroinflammation [1]. Although the initial inflammatory response mediated by microglia can be considered protective, excessive pro-inflammatory responses of microglia are increasingly recognized as part of pathogenesis [1,32,33]. Autophagy and inflammatory processes are reciprocally interconnected, intersecting at multiple points through key regulatory molecules of the canonical autophagy pathway such as p62/SQSTM1 [4,33,34,35]. Autophagy inhibition is generally correlated with higher levels of p62/SQSTM1, while autophagy activation is associated with lower protein levels. Additionally, p62/SQSTM1 can also be detected as puncta [34]. The p62/SQSTM1 protein participates directly in proteasomal degradation and regulates the formation of protein aggregates [9,36,37]. Since undegraded p62/SQSTM1 accumulates when autophagy is inhibited, and decreased levels of p62/SQSTM1 can be observed when autophagy is induced, p62/SQSTM1 may be used as a marker to study autophagic flux [11,38].

Autophagy can also intricately influence inflammation and microglial activation, suggesting that promoting the early stages of autophagy could be the basis for potential therapeutic approaches in diseases associated with neuroinflammation [33]. Several experimental factors can influence changes in p62/SQSTM1 levels [34,39]. Most studies reinforce the inhibitory effects of LPS-mediated microglial activation and demonstrate some inflammatory processes in the main aspects of autophagy [22,33,34]. As p62/SQSTM1 participates in complex regulatory systems and signaling pathways, autophagy is also regulated by interactions with pro- and anti-inflammatory mediators, the effects of which can be comprehensively interpreted [4,34]. For example, LPS inhibited autophagy and reduced rapamycin-induced autophagic flux in N9 microglial cells, as evidenced by increased levels of p62/SQSTM1 [33]. Furthermore, a recent study demonstrated that LPS-induced mitochondrial fission elevated p62/SQSTM1 expression [20]. Others have also made similar observations in hepatocytes [40]. However, under certain conditions, LPS has been shown to induce autophagy in microglia [20,41] and macrophages [42]. Thus, both the results and their interpretations present a complex and varied picture depending on experimental setups, investigation methods, timing, cell type/line, and other factors [22,33,34]. Therefore, the effects of compounds that affect inflammatory processes and microglial activation from the perspective of autophagy are not always straightforward [4,34].

Pharmacological modulators of autophagy have been widely used in basic research and preclinical studies. BAF and chloroquine are commonly used compounds that inhibit autophagy by targeting lysosomes but through different mechanisms [26]. BAF is a potent inhibitor of autophagocytosis and cell proliferation [43,44,45,46,47]. BAF inhibits autophagy only in the phagosome-lysosome fusion stage. Therefore, when administered concurrently with autophagy-inducing agents, it reveals their autophagy-promoting effects by preventing the rapid fusion/degradation that normally follows the accelerated phagosome production (which is inhibited by BAF). During the quantification of autophagy, lysosomal inhibitors, such as BAF, play a critical role in preventing autophagosome degradation, thus allowing its accumulation to be observed [48]. In our study, pretreatment with BAF resulted in a significantly lower number of microglial cells in all cultures involved, similar to the results of previous studies on cancer cells [43,47]. This effect on proliferation is probably due to inhibition of autophagy by inhibiting vacuolar ATPase [43,47].

Various effects can cause transient changes that complicate the interpretation of autophagic flux based solely on protein measurement [34]. In a recent study, the inhibitory effect of RST on the autophagic process was studied in rat insulinoma cells [49]. They found that RST significantly increased p62/SQSTM1 expression and was interpreted as a result of the mTOR-dependent and autophagy-inhibiting effects of RST [49]. However, in another study, atorvastatin improved autophagy, as evidenced by down-regulation of p62/SQSTM1 levels [50]. We found a significant decrease in p62/SQSTM1-labeled puncta in RST-treated microglia as compared to controls, but a significant increase when pretreatment with BAF was used before the RST treatment. Western blot analysis of p62/SQSTM1 protein expression showed only some non-significant increase in the number of p62/SQSTM1-positive autophagosomes when BAF pretreatment was used. It should be noted that we used RST instead of atorvastatin, and discrepancies between the results may be attributed to the different mechanisms of action of these statins and the complexity of the autophagocytotic process. Further comprehensive experiments and mechanistic studies are warranted to elucidate these discrepancies.

In the present study, we observed a quite dramatic effect of BAF pretreatment on microglia survival, as all BAF-pretreated groups had significantly less microglial cells compared to their respective controls. BAF is well known to possess cytostatic and apoptosis-inducing properties [45,51], but to our knowledge, this is the first time such an effect on microglia survival has been reported. Furthermore, significant differences in Iba1 expression were observed in the groups treated with RST and LPS+RST with or without BAF pretreatment; a nonsignificant decrease was also detected between the LPS and BAF-LPS treated groups. The changes seen in Iba1 levels are probably the result of the complex effects of BAF pretreatment on cell growth and survival that extend to protein synthesis and metabolic and immunological signaling pathways. We also found only slightly elevated cytoplasmic levels of p62/SQSTM1 in both LPS-treated and RST-treated microglia. However, when cultures were treated simultaneously with LPS and RST, a significant accumulation of this protein was detected, probably due to the combined effects of the treatments. This could be due to inhibition of autophagy, as the degradative process of autophagy is blocked. Interestingly, the same treatments resulted in opposite regulation of autophagosome puncta formation, as both LPS and RST decreased (the latter significantly) the number of p62/SQSTM1-labeled puncta in microglia, indicating an additional altered state of autophagy. Furthermore, we observed distinct immunocytochemically detected responses in RST treated microglia when BAF pretreatment was administered. In particular, a significant decrease in p62/SQSTM1 immunopositive autophagosomes was observed in microglial cells, suggesting that complex signaling mechanisms and immunological interactions may be the basis for the modulation of autophagocytosis by both BAF and RST. The slight decrease seen in the number of autophagosomes after LPS treatment compared to controls is similar to that seen in BV2 cells [22], although this change was not significant, unlike in the group treated with RST. Our results corroborate previous findings on the impact of BAF on autophagy regulation. Furthermore, these findings provide additional evidence to support the role of BAF in modulating autophagic processes, highlighting its potential effects on cellular responses and autophagy-related pathways.

In addition to the conventional homeostatic and adaptive function of autophagy, recent studies have shown that autophagy also plays a critical role in immunity by regulating cytokine production and release, inflammasome activation, antigen presentation, and clearance of invading pathogens [52]. Studies have shown that autophagy induction could inhibit inflammation, especially in immune cells such as macrophages and dendritic cells. Recently, the enhancement of autophagy has been reported to improve the pathogenesis of multiple sclerosis or experimental autoimmune encephalomyelitis by limiting inflammation [53]. By participating in the effective elimination of cellular damage, autophagy also regulates neurodegenerative and aging processes [12,14,16,54]. In addition to neurofibrillary tangles and neuritic plaques, p62/SQSTM1 may also play an important role in neurodegenerative disorders [55]. Since autophagy is one of the major degradative pathways that cells use to achieve proteostatic balance, its activation appears especially promising in the potential treatment of these diseases [21].

Autophagy is fundamentally intertwined with cellular housekeeping processes, and its altered function can influence the expression of numerous proteins. In this study, we demonstrated suppression of autophagic activity, as evidenced by a significant increase in p62/SQSTM1 protein when LPS was added together with RST. Furthermore, pretreatment with BAF allowed us to perform a more precise evaluation of regulatory effects, particularly in response to LPS and RST treatments. This insight sheds further light on the intricacies of autophagy regulation in the context of inflammation and improves our understanding of the complex mechanisms that govern the modulation of autophagy in microglial cells.

## 4. Materials and Methods

### 4.1. Animals

All animal experiments were carried out in strict compliance with the European Council Directive (86/609/EEC) and the EC regulations (O.J. of EC No. L 358/1, 18/12/1986) regarding the care and use of laboratory animals for experimental procedures and followed the relevant requirements of Hungarian and local legislation. The experimental protocols were approved by the Institutional Animal Welfare Committee of the University of Szeged (II./1131/2018; date of approval: 30 May 2018). The pregnant Sprague–Dawley rats (170–190 g; one animal per cage) were kept under standard housing conditions and fed ad libitum. Five breeding runs (4–6 pregnant rats each) provided the litters (6–12 pups from each mother) from which independent culturing experiments were performed.

### 4.2. Reagents and Antibodies

Lipopolysaccharide (LPS; Sigma-Aldrich, St. Louis, MO, USA) was used as an immunochallenge (20 ng/mL in final conc.), while RST (Sigma-Aldrich) was used as an anti-inflammatory agent (1 μM in final conc.). A stock solution of BAF (Sigma-Aldrich; 160 μM) was used to treat the cultures (50 nM in final conc.) to inhibit autophagy. Antibodies used in our immunohistochemical and Western blot studies are listed in Table 1. An antibody against the ionized calcium-binding adaptor molecule 1 (Iba1), an intracellular Ca^2+^-binding protein, was used to detect microglia [56]. An antibody against the p62/SQSTM1 was used to label autophagosomal vesicles [14,54] in Iba1-positive microglial cells. The anti-glyceraldehyde 3-phosphate dehydrogenase (GAPDH) antibody was used as an internal control in Western blot experiments [57]. Various dilutions of primary and secondary antibodies, incubation times, and blocking conditions were carefully tested for both immunohistochemistry and Western blot analysis. To detect the specificities of the secondary antisera, omission control experiments (staining without the primary antibody) were also performed. In such cases, no fluorescent or Western blot signals were detected.

### 4.3. Maintenance and Treatment of Cell Cultures

The cortices of neonatal rats were quickly dissected, minced, dissociated in 0.25% trypsin for 10 min at 37 °C, and incubated in 9 mL of Dulbecco’s Modified Eagle’s Medium (DMEM) containing 1 g/L D-glucose, 110 mg/L Na-pyruvate, 4 mM L-glutamine, 3.7 g/L NaHCO_3_, 10,000 U/mL penicillin G, 10 mg/mL streptomycin sulfate, and 25 μg/mL amphotericin B and 15% heat-inactivated fetal bovine serum (FBS; Thermo Fisher Scientific, Waltham, MA, USA). After centrifugation at 1000 g at room temperature (RT) for 10 min, the pellet was resuspended, washed in 10 mL of DMEM containing 10% FBS, and again centrifuged for 10 min at 1000 g and RT. The final pellet was filtered through a sterile filter (100 µm pore size; Greiner Bio-One Hungary Kft., Mosonmagyaróvár, Hungary) to remove tissue fragments that had resisted dissociation. Cells were resuspended in 2 mL of the same solution and then seeded on poly-L-lysine-coated culture flasks (75 cm^2^; 10^7^ cells/flask) and cultured at 37 °C in a humidified air atmosphere supplemented with 5% CO_2_. The medium was changed the next day and then the fourth day. After 7 days of culture, microglial cells in primary cultures were shaken off using a platform shaker (120 rpm for 20 min) at 37 °C, collected from the supernatant by centrifugation (3000× *g* for 8 min at RT), resuspended in 4 mL of DMEM/10% FBS, and seeded in the same medium on poly-L-lysine-coated coverslips (15 × 15 mm; 2 × 10^5^ cells/coverslip) for immunocytochemistry or in poly-L-lysine-coated Petri dishes (10^6^ cells/Petri dish) for Western blot analysis. The number of cells collected was determined in a Bürker chamber after trypan blue staining.

On subDIV6, the expanded microglia-enriched cultures were divided into two sets of cultures. Each set included (1) control (unchallenged), (2) LPS-treated (immunochallenged with 20 ng/mL LPS in final conc., dissolved in DMEM), (3) RST-treated (1 μM final conc., dissolved in DMEM), and (4) LPS+RST-treated cultures (20 ng/mL LPS and 1 μM RST in final concentrations). LPS, RST, and LPS+RST treatments lasted for 24 h. The first set of cultures was without BAF treatment, while the cultures in the second set received a 3-h pretreatment with BAF (50 nM in final conc., diluted from a 160 μM stock solution in DMEM) for each experimental culture. Specifically, the following eight culture types were used: (1) control cultures (unchallenged and untreated), (2) LPS-challenged cultures; (3) RST-treated cultures, (4) LPS-challenged+RST-treated cultures, (5) BAF-pretreated control cultures, (6) BAF-pretreated+LPS-challenged cultures, (7) BAF-pretreated+RST-treated cultures, and (8) BAF-pretreated+LPS-challenged+RST-treated cultures.

### 4.4. Determination of Microglia Cell Purity and Survival

For the determination of microglial cell purity, DAPI-labeled cell nuclei that belonged to Iba1-immunopositive cells were counted on coverslips in cultures (subDIV7). For each culture, at least 50 randomly selected microscope fields were analyzed. Cultures typically had 73% purity for microglia from independent culture experiments [58], but we selected and used cultures only with ~99% purity for these studies (approximately one in seven attempts). For the determination of microglial cell survival, fluorescent light microscopic pictures were taken from each independent experiment at 20 × magnification. The number of Iba1-positive microglial cells in a field of view was counted in 15 images with the “cell counter” plugin of the software program ImageJ and averaged (± SD).

### 4.5. Western Blot Analysis

Secondary microglia (subDIV7) were collected, homogenized in 50 mM Tris–HCl (pH 7.5) containing 150 mM NaCl, 0.1% Nonidet P40, 0.1% cholic acid, 2 μg/mL leupeptin, 1 μg/mL pepstatin, 2 mM phenylmethylsulfonyl fluoride, and 2 mM EDTA, and centrifuged at 10,000× *g* for 10 min at 4 °C. The pellet was discarded, and the concentration of soluble protein of the supernatant was determined [59]. Protein content (5–10 μg) was separated on a sodium dodecyl sulfate (SDS)–polyacrylamide gel (4–10% stacking gel/resolving gel), transferred to Hybond-ECL nitrocellulose membrane (Amersham Biosciences, Little Chalfont, Buckinghamshire, England), blocked for 1 h in a 5% solution of non-fat dry milk in Tris-buffered saline (TBS) containing 0.1% Tween-20, and incubated for overnight with the appropriate primary antibodies (Table 1) as well as the mouse anti-GAPDH monoclonal antibody serving as internal control. After five washes in 0.1% TBS–Tween-20, the membranes were incubated for 1 h with the appropriate peroxidase conjugated secondary antibodies (Table 1) and washed three times. The enhanced chemiluminescence method (ECL Plus Western blot detection reagents; Amersham) was used to reveal immunoreactive bands according to the manufacturer’s protocol. The protocols were optimized for each antibody with respect to epitope accessibility, polyacrylamide gel separation, antibody dilution, and chemiluminescence signal intensity (exposure time, film development, etc.).

Digital grayscale images of immunoblots were acquired by scanning the autoradiographic films with a desktop scanner (Epson 430 Perfection V750 PRO; Seiko Epson Corp., Suwa, Japan). The images were scanned and processed at identical settings to allow comparisons of the blots from different samples. The bands were outlined and analyzed by densitometry using the ImageJ computer program (version 1.47; developed at the U.S. National Institutes of Health by W. Rasband [60], available at https://imagej.net/Downloads; accessed on 10 July 2013). Integrated optical density values for Iba1 and p62/SQSTM1 immunoreactivities were calculated as the percentage of control values that had been normalized to the signal of the internal standard GAPDH.

### 4.6. Immunocytochemistry

For multicolor fluorescent immunocytochemistry, secondary microglia-enriched cultures (subDIV7) on poly-L-lysine-coated coverslips were used [56]. The cells were fixed in 4% formaldehyde in 0.05 M PBS (pH 7.4 at RT) for 5 min then rinsed in 0.05 M PBS for 3 × 5 min. After permeabilization and blocking of nonspecific sites in 0.05 M PBS solution containing 5% normal goat serum (Sigma-Aldrich) and 0.3% Triton X-100 for 60 min at 37 °C, cells on the coverslips were incubated overnight at 4 °C with the appropriate primary antibody (Table 1) in 1% heat-inactivated bovine serum albumin (Sigma-Aldrich) and 0.3% Triton X-100. Cultured cells were washed for 3 × 5 min at RT in 0.05 M PBS then incubated with the appropriate secondary antibody conjugated to Alexa Fluor fluorochrome (Table 1) in the above solution, but without Triton X-100, in the dark for 2 h at RT. Cells on the coverslip were washed for 3 × 5 min in 0.05 M PBS at RT, and nuclei were stained in a 0.05 M PBS solution containing 1 mg/mL polyvinylpyrrolidone and 0.5 μL/mL 2-[4-(aminoiminomethyl)phenyl]-1H-indole-6-carboximidamide hydrochloride (DAPI; Thermo Fisher Scientific). The coverslips were then rinsed in distilled water for 5 min, air-dried, and mounted on microscope slides in Vectashield mounting medium (Vector Laboratories, Burlingame, CA, USA).

### 4.7. Image Analysis

Digital images of the cultured microglia were captured on a Leica DMLB epifluorescence microscope using a Leica DFC7000 T CCD camera (Leica Microsystems CMS GmbH, Wetzlar, Germany) and the LAS X Application Suite X (Leica). A total of 1350 Iba1-immunopositive microglia from control and treated cultures were analyzed for this study (for individual cells, see their cell ID in the Supplementary Table S2). To identify Iba1-immunopositive microglial cells and their p62/SQSTM1-positive autophagosomal puncta formations, the computer program ImageJ was used. Iba1-positive microglia from ten randomly sampled microscope fields were counted using the ImageJ cell counter plugin for each of the eight culture types analyzed. For the quantitative analysis of p62/SQSTM1 immunopositive phagosomes, three independent immunocytochemical experiments were performed. From each group (one control group and seven experimental groups with or without BAF pretreatment), one hundred Iba1-positive microglial cells were analyzed for specific p62/SQSTM1 immunoreactive puncta formation [61] in twenty microscope fields of view randomly sampled from three coverslips and counted using the computer program ImageJ [56,62].

**Figure 6:**
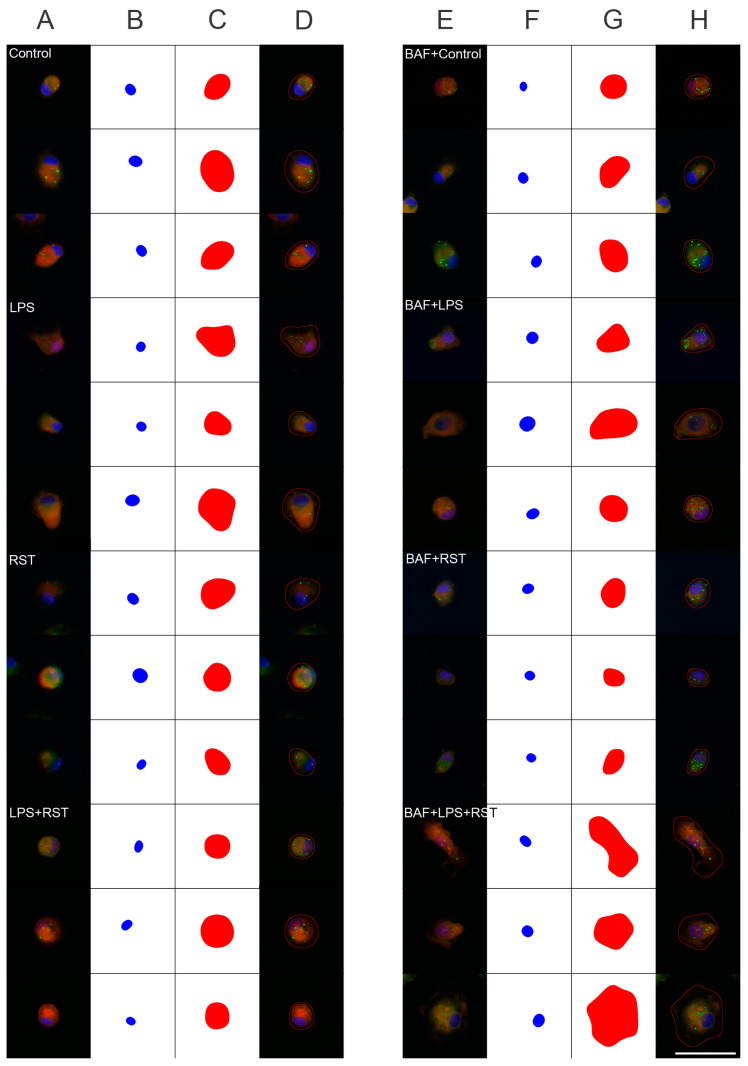
Digital image processing and measurement pipeline for cell segmentation and autophagosome detection. This figure illustrates representative examples of cell segmentation and autophagosome detection according to the measurement protocol described in the Materials and Methods section. Each microglia is depicted with four images: (**A**,**E**) the original image, followed by masks representing (**B**,**F**) the cell nucleus (blue) and (**C**,**G**) the cell contour/cytoplasm (red). The final image (**D**,**H**) overlays these masks on the original image, highlighting the autophagosomes (green). Three sample images are provided for each group (Control, LPS, RST, LPS+RST, BAF+Control, BAF+LPS, BAF+RST, BAF+LPS+RST), offering illustrative examples of the process. The following microglia, identified by their cell IDs (Supplementary Table S2), are displayed: Controls: 145, 197, 209; LPS: 221, 368, 374; RST: 493, 497, 502; LPS+RST: 648, 663, 733; BAF+Control: 737, 775, 795; BAF+LPS: 876, 896, 912; BAF+RST: 1022, 1045, 1050; BAF+LPS+RST: 1225, 1229, 1282. Scale bar (for all pictures): 50 μm.

The image processing and measurement pipeline, developed specifically for this study, is depicted in Figure 6. Briefly, digital images in tagged image file formats (.tif) were opened in ImageJ. For the quantitative analysis of p62/SQSTM1 immunopositive puncta the following eight consecutive image processing and measurement steps were performed.

(1) During scaling, software-recorded scale and the “Line selection tools” command in ImageJ were used to establish absolute dimensions in the study: 1 μm was determined to be 5.62 pixels.

(2) Since the diameter range of autophagosomes in mammals is 0.5–1.5 μm, these numbers were used both during the calculation of the area of the object and during the background correction, assuming a model of spherical objects. The area was calculated using the formula A = r^2^π = (d^2^π)/4 (where A = area, r = radius, d = diameter).

(3) During multichannel image analysis, the three-channel images were divided into channels using the command “Image => Color => Split Channels”. Autophagosomes were identified on the basis of signals from the green channel. Only signals within clearly defined contours in the processed red channel, corresponding to a specific cell, were considered, and the number of autophagosome puncta per cell was detected.

(4) The segmentation of the cells and the cell nuclei was implemented as follows. Segmented contours of Iba1-immunopositive cells were obtained through a multistep, fully automated, ImageJ command-based process. These contours were used in subsequent steps to determine the cytosolic autophagosome signals per cell. Complex multistep image processing was performed under identical settings for each image, ensuring that continuous, closed contours of individual microglial cells were extracted from microscopic photographs (.tif files). The 8-bit images (separate blue and red channels) were subjected to noise reduction, background correction, and thresholding in several steps. The extracted binary silhouettes were subjected to further image processing steps (such as additional noise reduction, various binary morphological operations, etc.). Automated exclusion (based on ImageJ macros) was applied to mutually exclude cell debris without a nucleus and nuclei enclosed by the cytoplasm without adequate Iba1 staining. In pursuit of refining the measurement, preprocessed but yet unthresholded 8-bit blue-channel images were also used for precise identification of individual nuclear signals. The peaks identified in the images were further filtered (to align with identifiable cell and nuclear signals), and segmenting lines created from the remaining peaks were used to separate adherent cells/cell nuclei in threshold-processed cell/nuclei images, facilitating the determination of individual cell contours (Process => Find Maxima… => Output type => Single Points/Segmented Particles).

(5) For the segmentation of autophagosome signals, a rolling ball algorithm with a radius of 4.215 pixels was applied during background subtraction in the green channel images, corresponding to 0.75 μm (1.5/2 μm) to eliminate background noise (in some cases, more diffuse cytosolic staining was present). Subsequently, a consistent threshold was established for each image and the “Watershed” and “Fill Holes” algorithms were applied to binarized images to extract uniquely identifiable autophagosome signals. As a result of the small and low-resolution nature of autophagosomes in the acquired images, temporary resizing of the images became necessary using the latter command as an alternative approach.

(6) Next, the exclusion range of the size of objects, identified as potential autophagosomes, was determined based on prior knowledge of the diameters of the autophagosomes. This range was converted to radii (0.25–0.75 μm) and then to pixels according to the established scale, resulting in values ranging from 1.405 to 4.215 pixels. The formula A = r^2^π = (d^2^π)/4 was applied (6.202–55.814), and the values obtained were rounded to integers to exclude size in the shapes in the images. Only objects within the 6 to 56 square pixels range were identified as potential autophagosome signals (Analyze => Analyze Particles => Show: Mask, Size (pixel^2^): 6–56).

(7) The exclusion of noncytosolic/non-microglial signals was determined as follows. When objects falling within areas enclosed by individual cell contours identified using the red (and blue) channel were focused, signals identified as potential autophagosomes were narrowed down. This step excluded non-cytosolic/non-microglial signals (potential artifacts or contamination).

(8) Finally, the remaining objects were specifically identified as autophagosome signals to determine the number of autophagosomes per microglial cell. This counting was performed individually per cell on the binarized, processed, and filtered images, executing the command within the unique cell contours (creating regions of interest from cell contours and placing them on the corresponding segmented green channel location: Analyze => Analyze Particles => Summarize => Count. Selected images showing the above image analysis pipeline are shown in Figure 6. Color correction and cropping of the light microscopic images were performed using Photoshop (Adobe Systems, Inc., San Jose, CA, USA) when photomicrographs were made for publication and assembled for a panel.

### 4.8. Statistical Analysis

All statistical comparisons were performed with GraphPad Prism 8.0 (GraphPad Software, San Diego, CA, USA). Western blot data were analyzed for statistical significance using one-way analysis of variance (ANOVA) with the Tukey post hoc test. The number of p62/SQSTM1 immunopositive puncta was analyzed using the Kruskal–Wallis test with the Dunn post hoc test using the Bonferroni adjustment. Values were presented as mean ± SD or as mean and median with 10–90 percentiles of the box plot. The significance level was established at *p* < 0.05.

## Figures and Tables

**Table 1:** Primary and secondary antibodies used in Western blot analysis and fluorescent immunocytochemistry.

Primary Antibody, Abbrev. Name	Primary Antibody, Full Name (Cat. No.)	Final Dilution	Company	Secondary Antibody, Full Name (Cat. No.)	Final Dilution	Company
Antibodies used in Western blot analyses						
Iba1	Rabbit anti-Iba1 polycl. ab. (019-19741)	1/1000	Abcam, Cambridge, UK	Anti-rabbit IgG, peroxidase conjug. (A-9169)	1/1000	Sigma, St. Louis, MO, USA
p62/SQSTM1	Mouse anti- p62 monocl. ab. (ab56416)	1/300	Abcam, Cambridge, UK	Anti-mouse IgG, peroxidase conjug. (A-9044)	1/1000	Sigma, St. Louis, MO, USA
GAPDH	Mouse anti-GAPDH monocl. ab. (G8795)	1/20,000	Sigma, St. Louis, USA	Anti-mouse IgG, peroxidase conjug. (A-9044)	1/1000	Sigma, St. Louis, MO, USA
Antibodies used in fluorescent immunocytochemistry						
Iba1	Rabbit anti-Iba1 polycl. ab. (019-19741)	1/1000	Abcam, Cambridge, UK	Alexa Fluor 568 goat anti-rabbit (A-11011)	1/1000	Invitrogen, Carlsbad, CA, USA
p62/SQSTM1	Mouse anti- p62 monocl. ab. (ab56416)	1/1000	Abcam, Cambridge, UK	Alexa Fluor 488 goat anti-mouse (A-11029)	1/1000	Invitrogen, Carlsbad, CA, USA

## Data Availability

The data are contained in the article.

## Supplementary Materials

The following supporting information can be downloaded at: https://www.mdpi.com/article/10.3390/ijms25158265/s1.

## Abbreviations

ANOVA	one-way analysis of variance
BAF	bafilomycin A1, ((3Z,5E,7R,8S,9S,11E,13E,15S,16R)-16-[(1S,2R,3S)-3-[(2R,4R,5S,6R)-2,4-dihydroxy-6-isopropyl-5-methyl-2-tetrahydropyranyl]-2-hydroxy-1-methylbutyl]-8-hydroxy-3,15-dimethoxy-5,7,9,11-tetramethyl-1-oxacyclohexadeca-3,5,11,13-tetraen-2-one)
DAPI	2-[4-(aminoiminomethyl)phenyl]-1H-indole-6-carboximidamide hydrochloride
DIV	day(s) in vitro
DMEM	Dulbecco’s modified Eagle’s medium
FBS	fetal bovine serum
GAPDH	glyceraldehyde 3-phosphate dehydrogenase (EC 1.2.1.12)
Iba1	ionized calcium-binding adaptor molecule 1
LPS	lipopolysaccharide
p62	ubiquitin-binding protein, 62 kDa (also known as sequestosome 1 (SQSTM1)
PBS	phosphate-buffered saline
rpm	revolutions per minute
RST	rosuvastatin, ((E)-7-[4-(4-Fluorophenyl)-6-isopropyl-2-[methyl(methylsulfonyl)amino]pyrimidin-5-yl]-(3R,5S)-3,5-dihydroxyhept-6-enoic acid)
RT	room temperature
SD	standard deviation
SDS	sodium dodecyl sulfate
SQSTM1	autophagosome cargo protein sequestosome 1 (also known as ubiquitin-binding protein, 62 kDa (p62))
subDIV	subcloned day(s) in vitro
TBS	Tris-buffered saline
