# Supplementary figures and images for: Bafilomycin 1A Affects p62/SQSTM1 Autophagy Marker Protein Level and Autophagosome Puncta Formation Oppositely under Various Inflammatory Conditions in Cultured Rat Microglial Cells
Source: Int J Mol Sci. 2024 Jul 29;25(15):8265. doi: 10.3390/ijms25158265 (PMC11311604; doi:10.3390/ijms25158265)

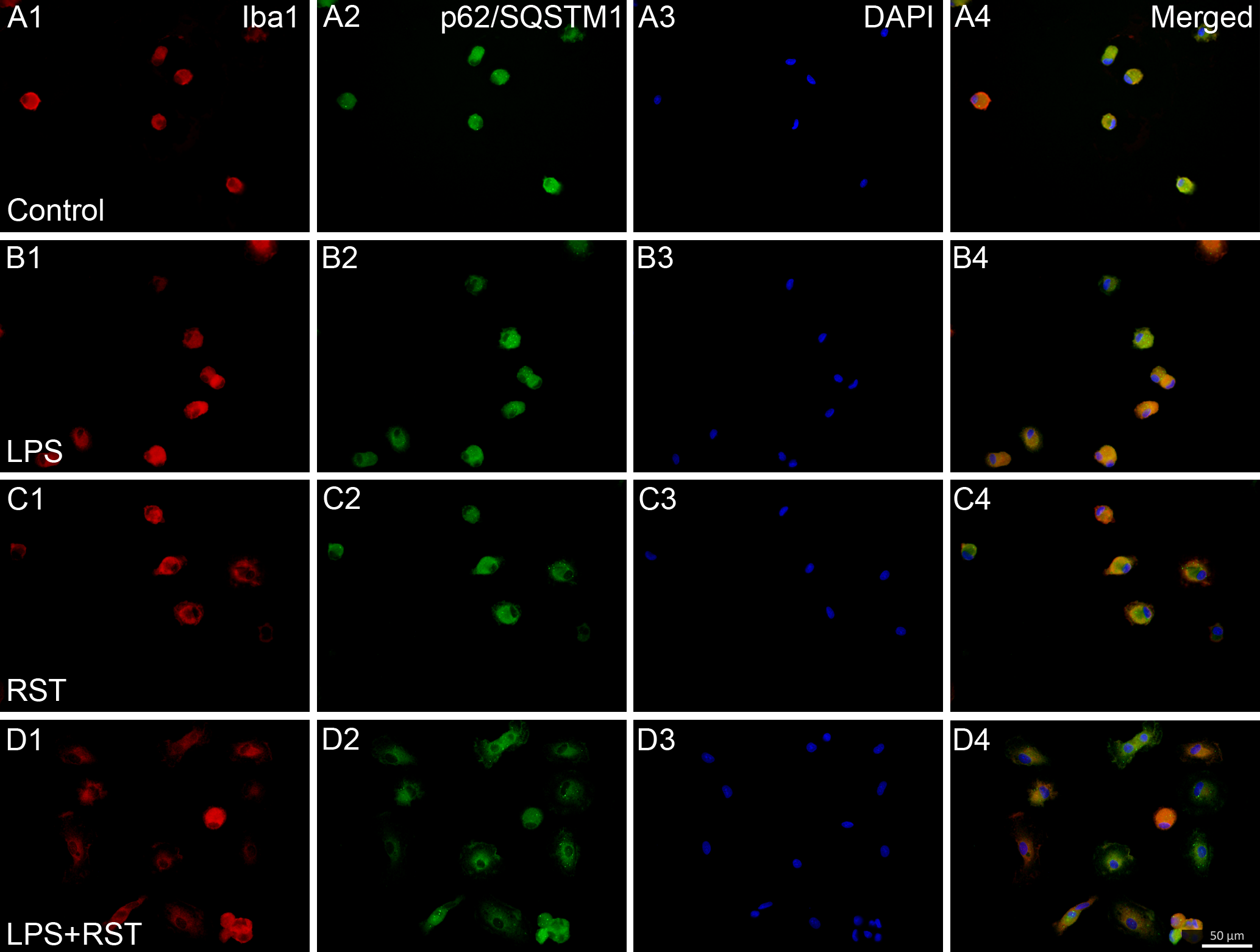

Supplement: Supplementary file 1 [file ijms-25-08265-s001.zip › Supplementary Figure S1.tif]

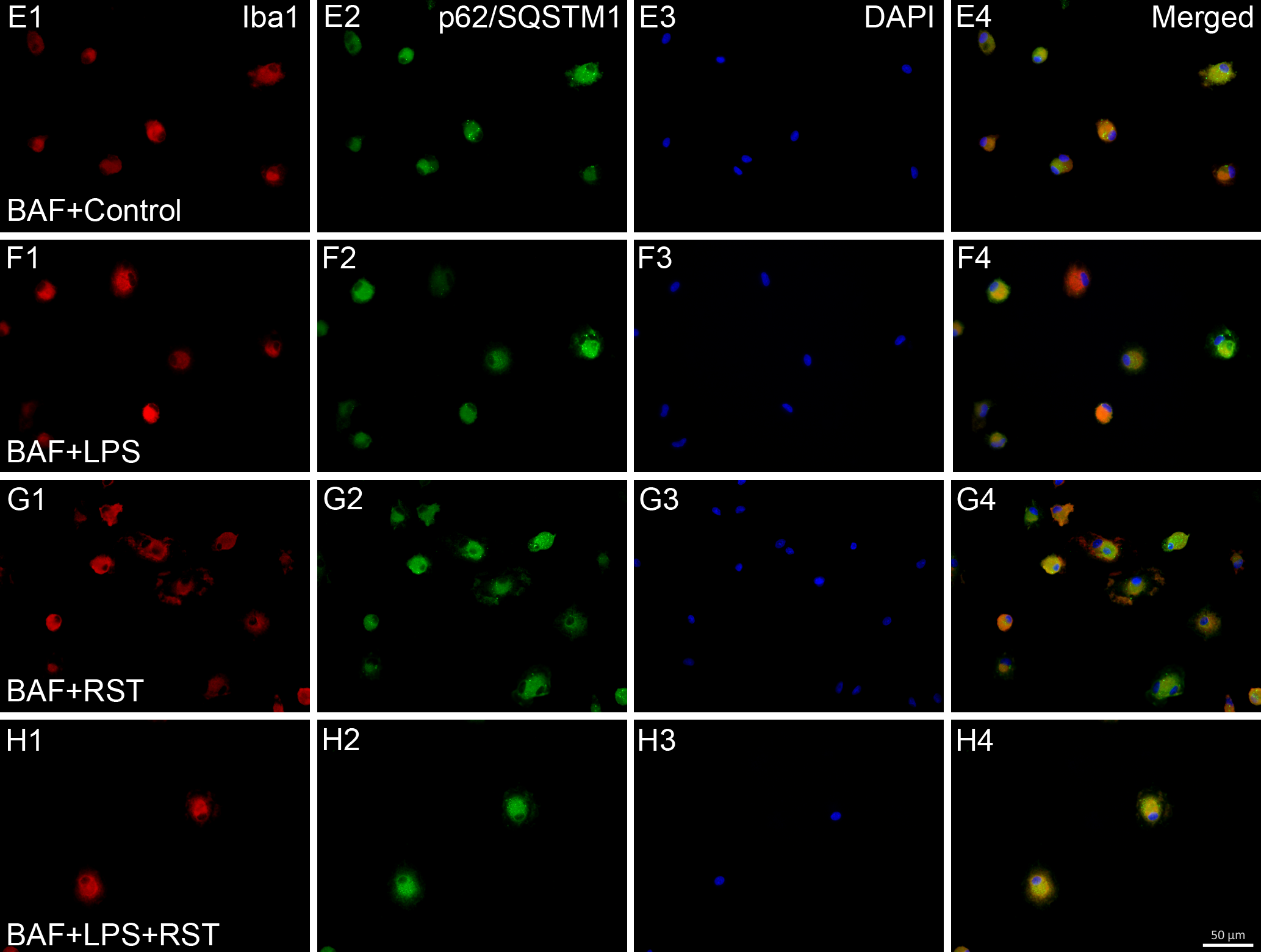

Supplement: Supplementary file 1 [file ijms-25-08265-s001.zip › Supplementary Figure S2.tif]
